# Cytomegalovirus esophagitis developing during chemoradiotherapy for esophageal cancer: two case reports

**DOI:** 10.1186/s13256-016-0947-y

**Published:** 2016-09-21

**Authors:** Kumiko Umemoto, Yasushi Kojima, Naoyoshi Nagata, Chizu Yokoi, Toshiyuki Sakurai, Masao Kobayakawa, Toshihiko Iizuka, Toru Igari, Mikio Yanase, Junichi Akiyama

**Affiliations:** 1 Department of Gastroenterology, National Center of Global Health and Medicine, 1-21-1 Toyama Shinjuku-ku, Tokyo, 162-8655 Japan; 2 Departments of Clinical Pathology, National Center of Global Health and Medicine, 1-21-1 Toyama Shinjuku-ku, 162-8655 Tokyo, Japan

**Keywords:** Cytomegalovirus, Esophagitis, Esophageal cancer, Case report

## Abstract

**Background:**

It is well known that cytomegalovirus esophagitis occurs in immunosuppressed patients. However, few reports have described cytomegalovirus esophagitis occurring during chemoradiotherapy for esophageal cancer.

**Case presentation:**

We report two cases of patients with cytomegalovirus esophagitis that developed during chemoradiotherapy for esophageal cancer. Cytomegalovirus esophagitis was diagnosed based on the presence of intranuclear inclusions in tumor biopsy specimens. The two Japanese patients presented with anorexia and fever, which improved with anti-cytomegalovirus treatment, and intranuclear inclusions were no longer seen in the specimens.

**Conclusions:**

The possibility of cytomegalovirus esophagitis must be kept in mind for patients with esophageal cancer presenting with prolonged fever or digestive symptoms while receiving chemoradiotherapy.

## Background

Cytomegalovirus (CMV) usually remains latent, but reactivation of the virus can occur in immunocompromised patients who have undergone bone marrow transplantation, have human immunodeficiency virus (HIV) infection, or are receiving immunosuppressant agents [1]. Under these circumstances, active infection may occur in many organs, particularly in the gastrointestinal tract (GIT), including the esophagus and colon [2, 3]. Patients with CMV infection of the GIT may manifest symptoms such as nausea, vomiting, chest pain, dysphagia, anorexia, and fever.

Diagnosis of CMV infection is made by endoscopic biopsy [4]. Detection of CMV antigenemia may support the diagnosis, but testing is not sufficiently sensitive and may yield false-negative results. While CMV infection is not rare in patients with HIV infection or those who have received stem cell transplantation, few reports have described cases of CMV esophagitis developing during chemoradiotherapy for esophageal cancer. We report herein two cases of patients with CMV esophagitis that developed during chemoradiotherapy for esophageal cancer in Japan.

## Case presentation

The clinical characteristics of the two Japanese patients are summarized in Tables 1 and 2.

**Table 1:** Patient characteristics

Case	Age	Sex	Stage	Regimen	Symptoms	Blood culture	Other infection
1	77	M	IIIC	FP + radiation	Fever, nausea, anorexia	No growth	-
2	61	M	IIIC	FP + radiation	Anorexia, fever	No growth	Pneumonia

*Abbreviations*: *M* Male, *IIIC* UICC 7th stageIIIC, *5-FU* 5-fluorouracil, *FP* 5-FU + cisplatin

**Table 2:** Clinical features

Case	At diagnosis						Antiviral therapy	Response to treatment		
	Intranuclear inclusions	CMV antigenemia	Endoscopic findings	WBC^a^ (/μL)	Lymphocytes^a^ (/μL)	CRP^b^ (mg/dL)		Symptoms	Intranuclear inclusions	CMV antigenemia
1							GCV, VGCV			
2	+	+	Ulcer, white base	2200	391	1.99	GCV, VGCV	Improved	Undetected	-

Clinical features at diagnosis of CMV esophagitis and after treatment with antiviral drugs. Low lymphocyte counts at diagnosis of CMV esophagitis are shown. The intranuclear inclusions disappeared, and the test for CMV antigenemia also became negative after the treatment

*Abbreviations*: *CMV* cytomegalovirus, *WBC* white blood cell, *CRP* C-reactive protein, *GCV* ganciclovir, *VGCV* valganciclovir

^a^The nadir WBC and lymphocyte counts before the diagnosis of CMV infection

^b^Serum CRP was measured at diagnosis of CMV esophagitis

### Case 1

**Fig. 1:**
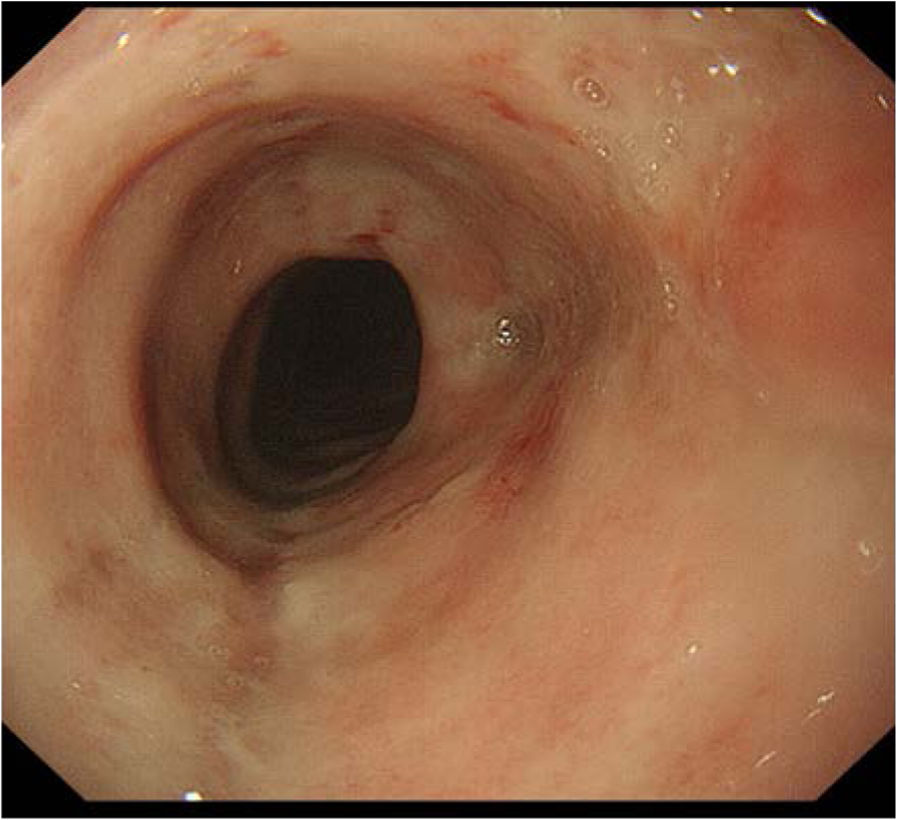
Upper gastrointestinal endoscopic findings in case 1. An ulcer with a white base is seen in the esophagus

**Fig. 2:**
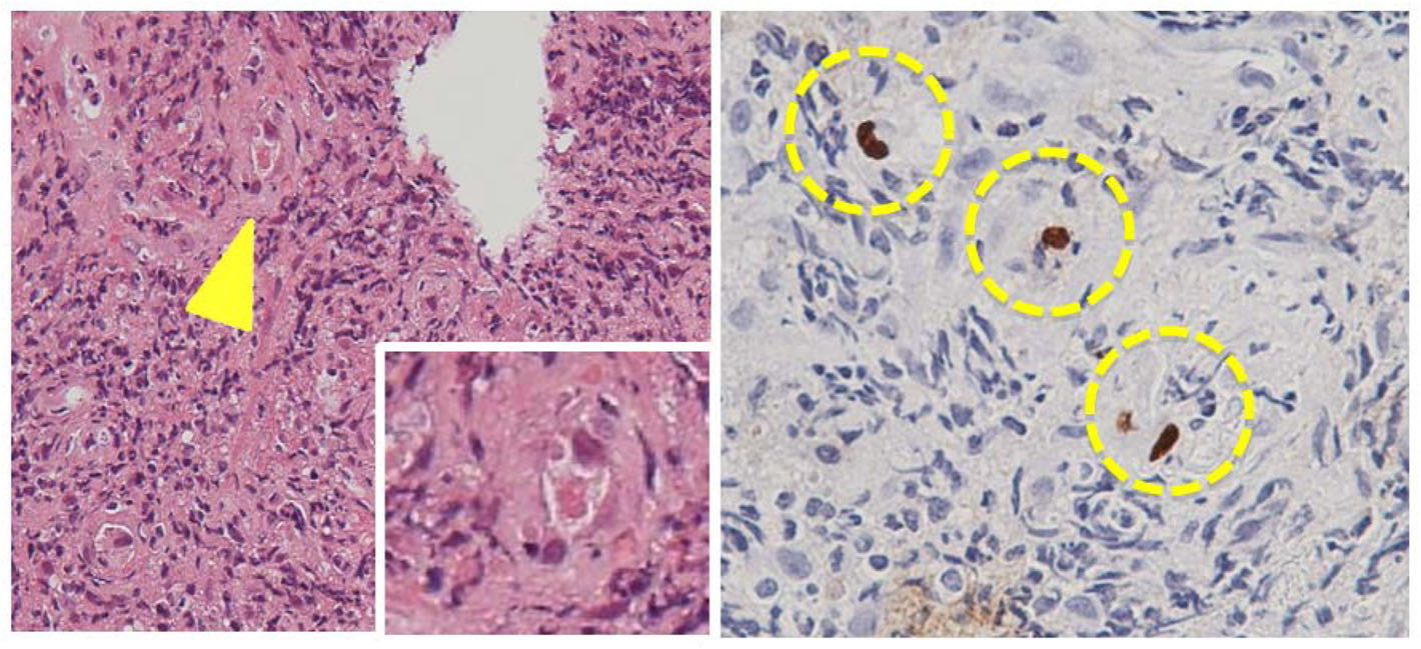
The arrow shows pathological findings of intranuclear inclusions obtained with biopsy at esophagus ulcer. There is the enlarged figure of the intranuclear inclusion on lower right box which suggest active esophagitis of CMV

**Fig. 3:**
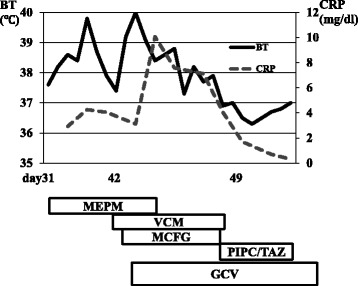
Clinical course from day 31 (after second course of FP) in case 1. *Abbreviations*: *MEPM* meropenem, *VCM* vancomycin, *MCFG* micafangin, *PIPC/TAZ* piperacillin/tazobactam, *GCV* ganciclovir, *VGCV* valganciclovir, *FP* 5-FU + cisplatin

A 77-year-old Japanese man with stage IIIC esophageal cancer was admitted to the hospital with fever (grade 1, Common Terminology Criteria for Adverse Events [CTCAE], version 4.0) and nausea (grade 2) after a second course of 5-fluorouracil (5-FU) (700 mg/m^2^/day, days 1–5 every 4 weeks) with concurrent radiotherapy (59.4 Gy/33 fr). His symptoms had shown no response to antibiotics or antifungal drugs. Nadir white blood cell (WBC) and lymphocyte counts before antiviral drug treatment were 820/μL (grade 4) and 180/μL (grade 4). CMV esophagitis was diagnosed based on the finding of intranuclear inclusions and positive CMV antibody immunostaining in a tumor biopsy, as well as a positive test result for CMV antigenemia. Findings on endoscopy at diagnosis are shown in Fig. 1 and pathological findings are provided in Fig. 2. Treatment with ganciclovir (GCV) (100 mg/kg/day) and then valganciclovir (VGCV) (450 mg every 2 days; reduced dose due to renal dysfunction) resulted in resolution of his fever and amelioration of his nausea (Fig. 3). Intranuclear inclusions were no longer detected in biopsy specimens from the tumor after treatment, and the test for CMV antigenemia also yielded negative results on day 43 after the completion of treatment.

### Case 2

A 61-year-old Japanese man with stage IIIC esophageal cancer presented with anorexia (grade 2) and fever (grade 1) after two courses of treatment with 5-FU (700 mg/m^2^/day, days 1–5 every 4 weeks) and cisplatin (70 mg/m^2^/day, day 1 every 4 weeks) with concurrent radiotherapy (60 Gy/30 fr). A lesion biopsy obtained after two courses of treatment revealed findings consistent with CMV esophagitis and serological testing for CMV antigenemia also yielded positive results. Nadir WBC and lymphocyte counts before antiviral pharmacotherapy were 2200/μL (grade 1) and 391/μL (grade 3). Treatment with VGCV (900 mg/day) followed by GCV (5 mg/kg/day) resulted in amelioration of his anorexia and fever. In addition, intranuclear inclusions and positive CMV antibody immunostaining were no longer evident in a lesion biopsy obtained after treatment, and test results for CMV antigenemia also became negative on day 128 after completion of antiviral treatment.

## Discussion

CMV esophagitis is a rare complication of chemoradiotherapy-induced myelosuppression, and is most commonly observed in patients who have undergone stem cell transplantation. Few reports in the literature appear to have described CMV infection of the GIT developing during chemoradiotherapy in patients with solid tumors [5].

In the cases reported here, CMV esophagitis was diagnosed based on examination of biopsy specimens obtained to evaluate the efficacy of anticancer regimens. In both Japanese cases, CMV esophagitis developed after two courses of chemotherapy (51–58 days after initiating treatment), and biopsy specimens were obtained from the site of the tumors, which were visualized endoscopically as ulcers with a white base. Test results for CMV antigenemia became negative after treatment with antiviral drugs in both cases. These patients manifested nausea or fever, and both symptoms and endoscopic findings improved with treatment. The present results suggest the utility of starting antiviral treatment for CMV esophagitis as soon as the diagnosis is established, rather than observation.

Diagnosis of CMV esophagitis based on endoscopic findings is difficult. The appearance of lesions is nonspecific, including ulcers and erosions in our cases, although a past report described specific features of CMV, such as irregular map-like erosions separate from tumor lesions [5]. CMV infection is confirmed mainly by immunostaining of tissue specimens obtained from the site of infection, and we recommend taking a lesion biopsy and indicating clinical suspicion of CMV infection to the pathologist, to ensure additional processing of the specimens, particularly for patients presenting with fever or digestive symptoms. A positive test result for CMV antigenemia may also support the diagnosis of CMV esophagitis by permitting rapid detection of CMV proteins in peripheral blood leukocytes, but the positivity rate at onset of CMV gastroenteritis is reported as only 50 % [6].

CMV infection in patients with esophageal cancer may be underreported, because fever and gastrointestinal symptoms in such patients may not be attributed to CMV infection, and instead simply managed as symptoms of radiation esophagitis or side effects of chemoradiotherapy. Severe lymphocytopenia as well as low WBC counts and neutropenia may lead to CMV esophagitis during chemoradiotherapy [7, 8].

## Conclusions

We have reported two rare cases of patients with CMV esophagitis that developed during chemoradiotherapy for esophageal cancer. Physicians and pathologists should keep the possibility of CMV esophagitis in mind for patients with esophageal cancer presenting with prolonged fever or digestive symptoms while receiving chemoradiotherapy. Biopsy of the involved tissues is essential for the diagnosis of CMV infection and antiviral treatment should be instituted once the diagnosis is established.

## Consent

Written informed consent was obtained from the patients for publication of this case report and any accompanying images. A copy of the written consent is available for review by the Editor-in-Chief of this journal.
